# Study on microstructure of 42CrMo steel by ultrasonic surface rolling process

**DOI:** 10.1038/s41598-023-48697-y

**Published:** 2023-12-02

**Authors:** Haojie Wang, Xiaoqiang Wang, Yingjian Tian, Yuanfei Ling

**Affiliations:** School of Mechatronics Engineering, Henan University of Science and Technology, Luoyang, 471003 China

**Keywords:** Mechanical engineering, Theory and computation

## Abstract

To explore the microstructure formation mechanism of 42CrMo steel under the strengthening of ultrasonic surface rolling process (USRP), the combination of theoretical analysis and experiment was used to conduct in-depth research on USRP. Firstly, according to contact mechanics and Hertz contact theory, the calculation model of contact stress distribution and elastoplastic strain between the rolling ball and the part during USRP is obtained. Secondly, the USRP processing test was carried out by single-factor experimental design method, and the microstructure of 42CrMo steel after USRP was analyzed by LEXT OLS5100 3D laser surface topography instrument and VEGA3 tungsten filament scanning electron microscopy, which found that with an increase in static pressure, residual stress and plastic strain gradually increase, the hardness firstly increases and then decreases, while surface roughness exhibits an initial decrease followed by an increase. The results show that USRP produces violent plastic deformation inside the material under the superposition of high-frequency impact and static pressure, at the same time, it refines the grains, so as to improve the surface performance of the part and improve its fatigue resistance.

## Introduction

42CrMo steel is widely used in the manufacture of wind power bearing rings due to its unique wear resistance and high hardness^1–3^. However, at the same time, because the wind turbine bearing rings are in the harsh working environment in the field, under harsh working conditions such as cold, sand and tide, the surface is very prone to fatigue pitting^4^, wear^5^ and even fracture^6^ and other failure forms. This will adversely affect the normal operation of wind turbines bearing rings. In order to extend the service life of wind turbine bearing rings and improve fatigue performance. The parts are strengthened by the surface treatment processing methods. There are many methods of surface strengthening of parts, including surface modification^7^ and surface mechanical deformation strengthening^8^. Surface modification includes laser processing^9^ and electrochemical processing^10^. Physical or chemical machining methods are used to form new structures on the surface of the part, thereby improving surface performance. However, Electric Discharging Machining (EDM) requires the use of high-voltage discharges, resulting in higher energy consumption, which may lead to increased electricity costs. The electrochemical machining process involves the use of corrosive liquids, necessitating proper handling and disposal to avoid environmental issues. Additionally, electrochemical machining requires specialized equipment and specific electrolyte fluids, which can result in relatively higher equipment and material costs. Surface mechanical deformation enhancement includes ultrasonic shot peening^11^ and rolling extrusion^12^. Ultrasonic shot peening can significantly increase the residual compressive stress on the surface of the part. On the contrary, it tends to result in a significantly higher surface roughness for the component, without any improvement in its friction performance. And traditional rolling methods establish a work-hardened layer on the surface of the workpiece, effectively reducing its surface roughness to a considerable extent. However, the wear and tear on the rolling ball will increase, resulting in too high processing costs. Ultrasonic surface rolling process (USRP)^13–16^ combines the advantages of the above two processing methods, which can significantly reduce the surface roughness of parts, simultaneously, it generates a work-hardened layer inside it to form the residual compressive stress. With the application of USRP, the wear on the rolling ball is greatly diminished, resulting in a marked enhancement of the surface quality of the part. This reduction in surface roughness and defects significantly elevates the surface performance of the workpiece. At the same time, it has a simple structure and strong flexibility in terms of operation. It can be flexibly fixed on lathes, milling machines and even machine center. The surface strengthening of 42CrMo steel is carried out by the method of USRP, which can improve its friction performance while increasing its residual compressive stress and hardness, which can enhance the surface performance of components and substantially prolong their service life.

Ituarte et al.^17^ used vibrating ball polishing technology to strengthen martensitic aging steel, and found that residual compressive stress was formed on the surface after USRP, the material hardness could reach 630HV. However, it did not elucidate the reasons for the surface mechanism of USRP-enhanced components. Liu et al.^18^ established a mathematical model of USRP residual stress and plastic strain according to the theory of elasticity and contact mechanics, at the same time, the test of 18CrNiMo7-6 steel was carried out, and it was found that the residual stress, the plastic strain and their layer depth increased with the increase of the static pressure and amplitude. However, the mathematical model was only established for residual stress and plastic strain, the conditions for achieving smooth rolling of the rolling ball in USRP were not considered. Wang et al.^19^ studied the influence of process parameters on Ti-6Al-4 V alloy treated by USRP. The residual stress distribution of USRP was studied by simulation and experiment. The results showed that the depth thickness of the residual stress layer is 0.4 mm, and there is a trend of increasing first and then decreasing along the depth direction. The research on USRP strengthening of the Ti-6Al-4V alloy was carried out using simulation and experimental methods. However, In USRP, the elastoplastic deformation of the components and the distribution of residual stresses play a significant role. Understanding these factors can guide the optimization of surface performance in USRP and help determine the optimal parameters for the process. Wu et al.^20^ exploited USRP to strengthen 40Cr steel, which found that the residual compressive stress first increased and then decreased along the layer depth, where the layer depth at this time was 200 μm. However, as a low-alloy steel with relatively lower strength and hardness, 40Cr steel is more easily processed by USRP. In contrast, 42CrMo steel is a medium–high alloy steel with higher strength and hardness. This necessitates a reconfiguration of USRP process parameters to meet its elevated strength and wear resistance requirements. Luo et al.^21^ conducted processing experiments on Ti6Al4V using the USRP method. The results indicated that the surface roughness of the components after USRP treatment was 0.231 μm, and the hardened layer depth was 304.38 μm. However, it was not taken into account that USRP strengthening process is an elastic–plastic contact deformation process under the action of impact loads, and the elastic deformation portion in the contact area has not been thoroughly investigated. Zhu et al.^22^ carried out USRP processing experiments on 2024-T3 aluminum alloy. The results revealed that USRP can effectively improve the surface morphology of the components, reducing surface roughness by over 80%. Qin et al.^23^ performed USRP on EA4T steel, and found that the surface roughness of the samples after USRP treatment is significantly lower than that of the untreated samples, and the average residual compressive stress has increased by 4.3 times, the cumulative effect of plastic deformation during the USRP was not taken into account.

In summary, scholars have conducted extensive research on the surface processing of USRP strengthened parts. However, these studies have primarily focused on observing surface roughness or hardened layer thickness after USRP, often overlooking the changes in material surface’s elastoplastic deformation and stress distribution during the USRP process. Furthermore, as USRP is a specific case of Hertzian contact, achieving smooth rolling of the rolling ball in USRP is rarely addressed. On the other hand, the influence of static pressure on the microstructure in the USRP has not been fully understood. Therefore, it is necessary to delve into the mechanisms underlying surface enhancement in USRP through a combination of theoretical analysis and processing experiments.

In this paper, a theoretical study on the surface formation mechanism of USRP-enhanced components is conducted firstly, combining principles of contact mechanics and Hertzian contact theory. Secondly, processing experiments on 42CrMo steel using the USRP method are performed. Additionally, Confocal Laser Scanning Microscopy and scanning electron microscopy are employed to observe the microstructure after USRP, investigating the evolution of 42CrMo steel’s microstructure during the USRP process. This research is of significant importance for uncovering the enhancement mechanism of USRP.

## USRP theory

### The influence of the rolling ball on workpiece in USRP

**Figure 1:**
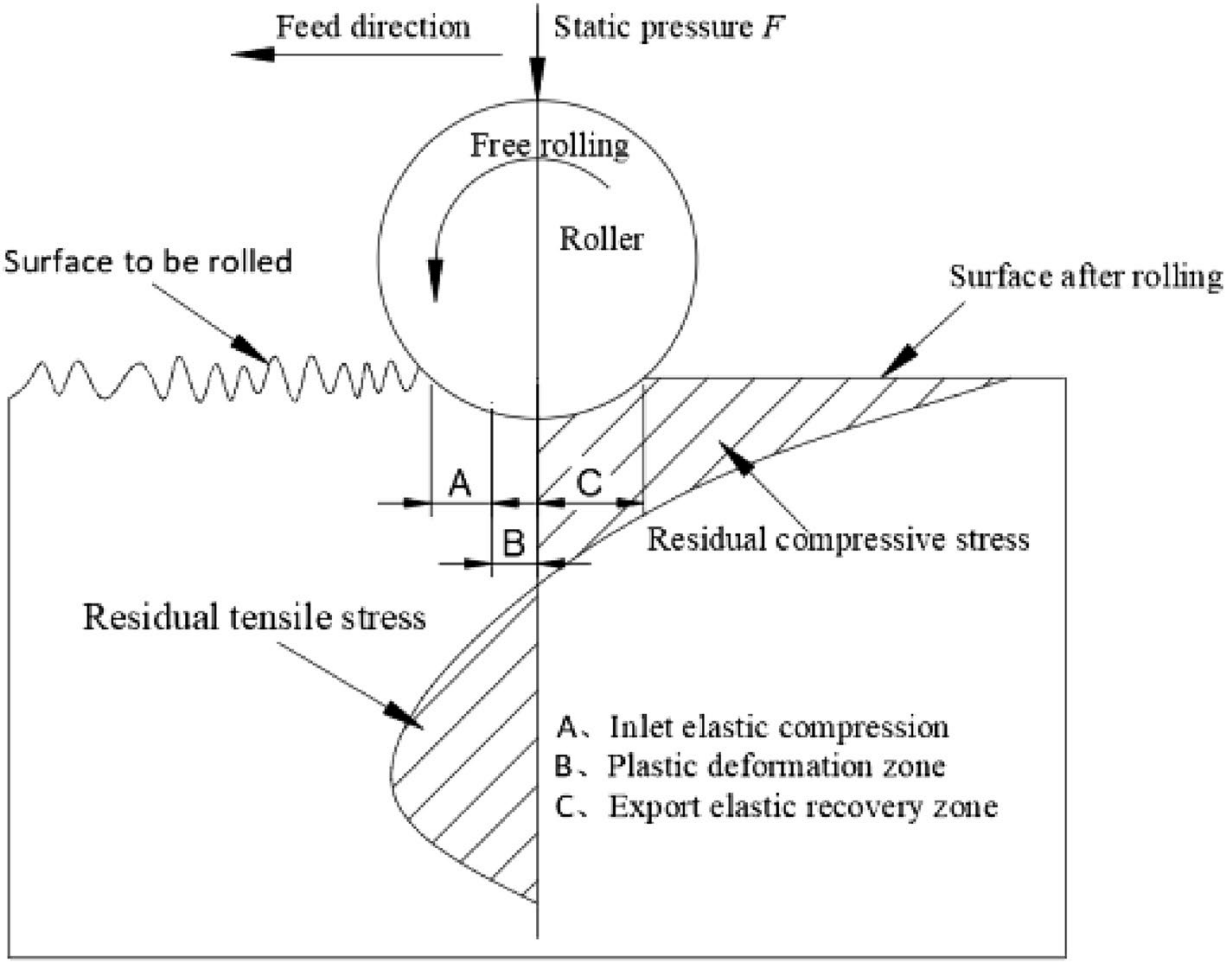
Processing principle of USRP.

Ultrasonic Surface Rolling Process (USRP)^24–26^ is a mechanical surface treatment process used to improve the surface properties of metallic materials, particularly the fatigue and wear resistance of components. This process is primarily applied to metals and alloys. It’s based on the principles of ultrasonic vibrations and plastic deformation. In USRP, a tool with a rolling ball shaped geometry is used. This tool is subjected to ultrasonic vibrations, typically at a high frequency. These vibrations are transmitted to the surface of the workpiece. The vibrations are typically in the ultrasonic range (typically above 20 kHz), which means they have a frequency higher than the audible range. At the same time, the ultrasonic vibrations cause the tool to exert cyclic compressive forces on the surface of workpiece. These cyclic forces lead to plastic deformation of the material in the surface layer. This deformation is localized and occurs within a shallow depth of the material. As shown in Fig. 1, under the combined action of static pressure applied by rolling balls and high-frequency vibration, it forms a work-hardened layer on the surface of the workpiece. In the contact region between the rolling ball and the specimen, the material of the specimen undergoes yielding, with a notable reduction in its yield limit value. This leads to plastic deformation transitioning from the surface towards deeper layers along the contour of the rolling ball. When the rolling ball disengages from the specimen’s surface, the surface metal experiences plastic deformation and retains this state. However, the flow rate of the sub-surface metal varies, resulting in non-uniform plastic deformation and a tendency to partially revert to its original shape. Nevertheless, this tendency is constrained by the surface material, which maintains the degree of plastic deformation. Consequently, the surface stress state of the specimen exhibits residual compressive stress. In other words, the internal elastic deformation zone is intended to recover from deformation, while the external work-hardened layer is designed to prevent further deformation, thereby converting the residual tensile stress left by machining into residual compressive stress. At the same time, USRP creates the effect of “peak shaving and valley filling” on the surface of the part, where it pushes the peaks left by machining into the valleys of the part's surface, facilitating metal flow. This process significantly reduces the surface roughness of the part. As a result of the plastic deformation, several beneficial effects are achieved, the cyclic plastic deformation causes a reduction in the size of the crystalline grains in the material’s microstructure. Smaller grains can enhance the material's mechanical properties. USRP imparts residual compressive stresses into the surface layer of the material. These compressive stresses are beneficial for enhancing fatigue resistance and preventing crack initiation. The plastic deformation leads to work hardening, making the surface layer of the material harder and more wear-resistant. The process can also lead to changes in the material's microstructure, such as increased dislocation density, which can further improve material properties. The resulting surface properties, including enhanced fatigue resistance and wear resistance, make USRP a valuable technique for extending the lifespan and performance of components subjected to cyclic loading or wear in various industries, including aerospace, automotive, and manufacturing.

### The influence of the rolling ball on workpiece in USRP

In the adjacent cycle, the feed movement is carried out at the same time as the tool head rolls, which can shave the surface of the specimen and fill the valley, refine the grain on the surface of the specimen, reduce the surface roughness of the specimen, and improve the surface hardness and residual stress of the specimen. In addition, this feed movement is not sliding friction, but rolling friction, which can greatly reduce the frictional heat generated by the machined surface and avoid the increase in wear of the tool head. The kinetic energy of the tool head can significantly reduce the axial stress on the surface of the specimen, reduce friction while inhibiting material accumulation in the place where the specimen is processed, thereby reducing the surface quality.

According to the theory of elasticity, the shear stress formula on a contact surface is shown in Eq. (1)^27^.

1 $$ \tau \left( r \right) = \tau_{0} \cdot \sqrt {1 - R_{2}^{2} /a^{2} } $$

where *τ* is the total shear stress, *τ*_0_ is the initial shear stress, *R*_*2*_ is the radius of the workpiece, *a* is the half width of contact area.

The shear displacement resulting from the contact surface is shown in Eq. (2)^28^.

2 $$ u_{x} = \frac{{\pi \tau_{0} }}{32Ga} \cdot \left[ {4\left( {2 - \mu } \right)a^{2} - \left( {4 - 3\mu } \right)x^{2} - \left( {4 - \mu } \right)y^{2} } \right] $$

where *u*_*x*_ is the shear displacement,* τ*_0_ is the initial shear stress,* G* is the shear modulus, *a* is the half width of contact area, *μ* is Poisson's ratio of 42CrMo steel, *a* is the half width of contact area, *x* is the distance along the X-direction, *y* is the distance along the Y-direction.

The solution of the rolling ball and the tangential problem of the material surface is based on the following condition: the stress distribution state of the rolling contact zone is superimposed by Hertz stress distribution of the surface displacement solution of two existing continuums. The stress distribution of the reinforced surface is shown in Eq. (3).

3 $$ \tau = \tau^{\left( 1 \right)} \left( x \right) + \tau^{\left( 2 \right)} \left( x \right) $$

where *τ* is the total shear stress distribution in Hertz contact, *τ*^(1)^ (*x*) is the shear stress of the rolling ball on the interface, *τ*^(2)^ (*x*) is the shear stress of the workpiece on the interface.

The stress states of the surfaces of the two continuums are shown in Eqs. (4) to (6).

4 $$ \tau^{(1)} (x) = \tau_{1} \cdot \left( {1 - \frac{{x^{2} }}{{a^{2} }}} \right)^{\frac{1}{2}} $$

5 $$ \tau^{(2)} (x) = - \tau_{2} \left( {1 - \frac{{\left( {x - d} \right)^{2} }}{{c^{2} }}} \right)^{\frac{1}{2}} $$

6 $$ d = a - c $$

where *τ*^(1)^ (*x*) is the shear stress distribution of the rolling ball, *τ*_1_ is the shear stress of the rolling ball, *x* is the distance along the X-direction, *a* is the half-width of the contact area, *τ*^(2)^ (*x*) is the shear stress distribution of the workpiece, *τ*_2_ is the shear stress of the workpiece, *d* is the difference between the half-width of the contact area and the half width of the front adhesion area,* c* is the half width of the front adhesion area, the total stress distribution of Hertz contact is shown in Eq. (7)^29^.

7 $$ p(x) = p_{0} \left( {1 - \frac{{x^{2} }}{{a^{2} }}} \right)^{\frac{1}{2}} $$

where *p* (*x*) is the total pressure distribution of Hertz contact, *p*_0_ is the pressure distribution, *x* is the distance along the X-direction, *a* is the half-width of the contact area.

**Figure 2:**
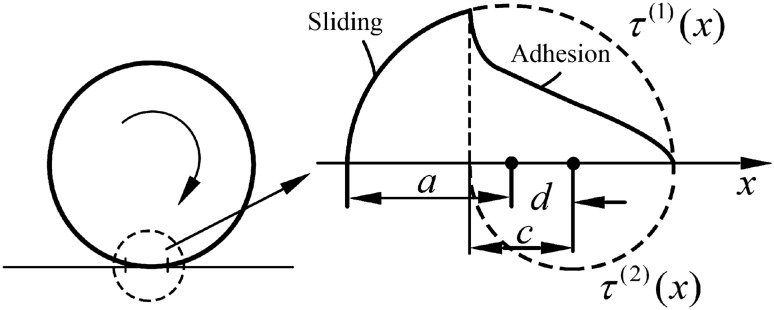
Schematic diagram of rolling contact.

As shown in Fig. 2, on the cross-section of the contact between the rolling ball and the material surface, the front-end position has begun to deform before reaching the contact, and when this part of the material element begins to contact the rolling ball, then the elements on both sides of the contact surface will not be relative displacement before leaving the adhesion zone. It can be inferred that the deformation is constant in the adhesion region, at the same time, Coulomb law of friction is met in the pure sliding region as shown in Eq. (8).

8 $$ \tau (x) = \mu \cdot p(x) $$

where *τ* (*x*) is the total shear stress distribution in Hertz contact, *μ* is Poisson's ratio of 42CrMo steel, *p* (*x*) is the total pressure distribution of Hertz contact.

The displacement caused by *τ*^(1)^ (*x*) and *τ*^(2)^ (*x*) is shown in Eqs. (9) and (10).

9 $$ u_{x}^{(1)} = C^{(1)} - \tau_{1} \frac{{x^{2} }}{{aE^{*} }} $$

10 $$ u_{x}^{(2)} = C^{(2)} + \tau_{2} \frac{{(x - d)^{2} }}{{cE^{*} }} $$

where *u*_*x*_^(1)^ is the displacement caused by the shear stress distribution of the rolling ball *τ*^(1)^ (*x*), *C* ^(1)^ is the displacement constant, *τ*_1_ is the shear stress of the rolling ball, *x* is the distance along the X-direction, *a* is the half-width of the contact area, E^*^ is equivalent elastic modulus, *u*_*x*_^(2)^ is the displacement caused by the shear stress distribution of the workpiece *τ*^(2)^ (*x*),* C* ^(2)^ is the displacement constant, *τ*_2_ is the second shear stress, *d* is the difference between the half-width of the contact area and the half width of the front adhesion area, *c* is the half width of the front adhesion area.

The partial differential form of the strain is shown in Eq. (11).

11 $$ \frac{{\partial u_{x} }}{\partial x} = - \tau_{1} \frac{2x}{{aE^{*} }} + \tau_{2} \frac{2(x - d)}{{cE^{*} }} $$

where ∂*u*_*x*_/∂*x* is the partial derivative of *u*_*x*_, *τ*_1_ is the shear stress of the rolling ball on the interface, *x* is the distance along the X-direction, *a* is the half-width of the contact area, E^*^ is equivalent elastic modulus, *τ*_2_ is the shear stress of the workpiece on the interface, *d* is the difference between the half-width of the contact area and the half width of the front adhesion area, *c* is the half width of the front adhesion area.

If the above smooth rolling conditions are met, then *τ*^(1)^ (*x*) is shown in Eq. (12).

12 $$ \tau_{1} = \mu \cdot p_{0} $$

where *τ*_1_ is the shear stress of the rolling ball on the interface, *μ* is Poisson's ratio of the 42CrMo steel, *p*_0_ is the pressure distribution.

If the above smooth rolling condition is met, the relationship between *τ*^(1)^ (*x*) and *τ*^(2)^ (*x*) is shown in Eq. (13).

13 $$ \tau_{2} = \frac{c}{a}\tau_{1} $$

where *τ*_2_ is the shear stress of the rolling ball on the interface, *c* is the half width of the front adhesion area, *a* is the half-width of the contact area, *τ*_1_ is the shear stress of the workpiece.

The strain in the adhesion zone is constant and can be obtained as Eq. (14).

14 $$ \frac{{\partial u_{x} }}{\partial x} = - \frac{{2\mu p_{0} d}}{{aE^{*} }} $$

where ∂*u*_*x*_/∂*x* is the partial derivative of *u*_*x*_,* μ* is Poisson's ratio of 42CrMo steel, *p*_0_ is the pressure distribution, *d* is the difference between the half-width of the contact area and the half width of the front adhesion area, *a* is the half-width of the contact area, E^*^ is equivalent elastic modulus.

The total transverse force on the contact surface at this point is shown in Eq. (15).

15 $$ F_{x} = \int_{ - a}^{a} {L\tau (x){\text{d}}x} = \left( {\frac{\pi }{2}a\mu p_{0} - \frac{c}{a} \cdot \frac{\pi }{2}c\mu p_{0} } \right)L = \mu F_{N} \left( {1 - \frac{{c^{2} }}{{a^{2} }}} \right) $$

where *F*_*x*_ is the total transverse force on the contact surface, *L* is the interfacial length,* τ* is the total shear stress of Hertz contact, *a* is the half-width of the contact area, *μ* is Poisson's ratio of 42CrMo steel, *p*_0_ is the pressure distribution, *c* is the half width of the front adhesion area, *F*_*N*_ is the positive pressure.

The radius of the adhesion zone is shown in Eq. (16).

16 $$ \frac{c}{a} = 1 - \frac{d}{a} = \left( {1 - \frac{{F_{x} }}{{\mu F_{N} }}} \right)^{\frac{1}{2}} $$

where *c* is the half width of the front adhesion area, *a* is the half-width of the contact area, *d* is the difference between the half-width of the contact area and the half width of the front adhesion area, *F*_*x*_ is the total transverse force on the contact surface, *μ* is Poisson's ratio of 42CrMo steel,* F*_*N*_ is the positive pressure.

Therefore, During the USRP process, the rolling ball imparts high-frequency impacts vertically to the material surface, and in the feed direction, motion involves smooth rolling rather than the sliding typically associated with conventional extrusion strengthening. This transition in the frictional behavior significantly reduces the heat generated by the frictional effects in the contact zone, preventing surface burns or rapid wear of the enhanced surface. The rolling action of the ball substantially reduces the lateral stress experienced by the material at the front end of the contact zone, lowering processing resistance and preventing the excessive accumulation of material at the front end of the contact zone, which could lead to a decline in surface quality.

### USRP contact mechanics analysis

The contact surface between the rolling ball and the cylindrical workpiece is elliptical. Therefore, according to the Hertz contact theory, the maximum contact stress when the two elastomers are in contact is shown in Eq. (17)^30^.

17 $$ P_{0} = \frac{3F}{{2\pi ab}} $$

where *P*_0_ is the maximum contact stress, *F* is the extrusion force, *a* is the major axis of the ellipse, *b* is the minor axis of the ellipse, so the contact stress of the arbitrarily shaped contact surface can be derived^31^, as shown in Eq. (18).

18 $$ q(x,y) = \frac{3F}{{2\pi ab}}\sqrt {1 - \left[ {\frac{{x^{2} }}{{a^{2} }} + \frac{{y^{2} }}{{b^{2} }}} \right]} $$

where *q*(*x*, *y*) is the contact stress of the arbitrarily shaped contact surface, *F* is the extrusion force, *a* is the major axis of the ellipse, *b* is the minor axis of the ellipse, *x* is the distance along the X-direction, *y* is the distance along the Y-direction.

Since the contact deformation is small relative to the size of the material itself, and the radius of curvature of the reinforced workpiece is much lower than the radius of the rolling ball, the impact pit generated by the single impact process can be approximately regarded as the rigid ball acting on the elastic surface, and the contact pit is approximately regarded as the circle. According to Hertz contact theory and elastic semi-infinite body theory, the calculation of the contact half-width in the contact deformation region is shown in Eq. (19).

19 $$ a_{1} = \sqrt {\frac{{4P_{1} R}}{{\pi E^{*} }}} $$

where *a*_1_ is the radius of the contact zone, *P*_1_ is the applied load, *R* is the radius of the rolling ball, E^*^ is the equivalent elastic modulus of the 42CrMo steel.

The calculation of the equivalent elastic modulus is shown in Eq. (20).

20 $$ E^{*} = E/\left( {1 - \mu^{2} } \right) $$

where E^*^ is the equivalent elastic modulus, *E* is the elastic modulus and *μ* is Poisson's ratio.

The distribution of contact stress in the contact area is shown in Eq. (21).

21 $$ p\left( x \right) = \frac{{2P_{1} }}{{\pi a_{1}^{2} }}\left( {a_{1}^{2} - x^{2} } \right)^{1/2} $$

where *p*(*x*) is the total pressure distribution of Hertz contact, *P*_1_ is the applied load, *a*_1_ is the radius of the contact zone, *x* is the abscissa.

The distribution of the first principal stress along the layer depth is shown in Eq. (22).

22 $$ \sigma_{x} = - \frac{{P_{0} }}{{a_{1} }}\left[ {\left( {a_{1}^{2} + 2z^{2} } \right)\left( {a_{1}^{2} + z^{2} } \right)^{ - 1/2} - 2z} \right] $$

where *σ*_*x*_ is the first principal stress along the layer depth, *P*_0_ is the maximum contact stress, *a*_1_ is the radius of the contact zone,* z* is the distance of the layer depth.

The distribution of the second principal stress along the layer depth is shown in Eq. (23).

23 $$ \sigma_{z} = - P_{0} a_{1} \left( {a_{1}^{2} + z^{2} } \right)^{ - 1/2} $$

where *σ*_*z*_ is the second principal stress along the layer depth, *P*_0_ is the maximum contact stress, *a*_1_ is the radius of the contact zone,* z* is the distance of the layer depth.

The distribution of principal shear stress along layer depth is shown in Eq. (24).

24 $$ \tau_{1} = P_{0} a_{1} \left[ {z - z^{2} \left( {a_{1}^{2} - z^{2} } \right)^{ - 1/2} } \right] $$

where *τ*_1_ is the principal shear stress along the layer depth, *P*_0_ is the maximum contact stress, *a*_1_ is the radius of the contact zone,* z* is the distance of the layer depth.

When the shape of the rolling ball is the sphere and the shape of the part is the cylinder, the machined contact surface in USRP can be regarded as an ellipsoidal pit that is projected vertically as an ellipse. It can be seen from the principle of USRP that the rolling ball continues to hammer the surface of the workpiece under the superposition of static pressure and high-frequency vibration, which causes plastic deformation on the surface of the test piece and forms the work hardening layer. The formula for calculating the major axis *a* and minor axis *b* in the ellipse of the contact surface is shown in Eq. (25).

25 $$ \left\{ \begin{gathered} a = \frac{{v_{x} }}{{2f_{r} }} \hfill \\ b = \sqrt {2R_{1} x_{1} - x_{1}^{2} } \hfill \\ \end{gathered} \right. $$

where *a* is the major axis in the ellipse of the contact surface, *v*_*x*_ is the velocity of the rolling ball perpendicular to the contact surface, *f*_*r*_ is the vibration frequency,* b* is the minor axis in the ellipse, *R*_1_ is the radius of the rolling ball, *x*_1_ is the extrusion depth of the rolling ball.

According to Eq. (25), the calculation of the projected area of the contact surface between the rolling ball and the workpiece in the radial direction in USRP is shown in Eq. (26).

26 $$ S = \pi ab = \frac{{\pi v_{x} }}{{2f_{r} }}\sqrt {2R_{1} x_{1} - x_{1}^{2} } $$

where* S* is the projected area of the contact surface between the rolling ball and the workpiece in the radial direction, *a* is the major axis in the ellipse of the contact surface,* b* is the minor axis in the ellipse, *v*_*x*_ is the velocity of the rolling ball perpendicular to the contact surface, *f*_*r*_ is the vibration frequency,* R*_1_ is the radius of the rolling ball, *x*_1_ is the extrusion depth of the rolling ball.

Similarly, the maximum pressure at the center of the contact surface is calculated as shown in Eq. (27)^32^.

27 $$ P_{0} = \frac{{3F_{d} }}{2S} = \frac{{3f_{r} \left[ {F - 4\pi^{2} mf_{r}^{2} A\sin \left( {2\pi f_{r} t} \right)} \right]}}{{\pi v_{x} \sqrt {2R_{1} x_{1} - x_{1}^{2} } }} $$

where* P*_0_ is the maximum pressure at the center of the contact surface, *F*_*d*_ is the dynamic force,* S* is the projected area of the contact surface between the rolling ball and the workpiece in the radial direction, *f*_*r*_ is the vibration frequency, *F* is the static pressure, *m* is the quality of rolling ball, *A* is the vibration amplitude,* t* is vibration time, *v*_*x*_ is the velocity of the rolling ball perpendicular to the contact surface,* R*_1_ is the radius of the rolling ball, *x*_1_ is the extrusion depth of the rolling ball.

The elastic deformation mainly occurs during USRP in the vertical direction of the contact surface between the rolling ball and the workpiece, and the elastic deformation in the three directions of *x*, *y* and *z* is related to the major axis *a* and minor axis *b* of the ellipse. The elastic stress in the three directions is shown in Eq. (28).

28 $$ \left\{ \begin{gathered} \sigma_{x} \left( x \right) = P_{0} \left( {1 + \frac{{x_{1}^{2} }}{ab}} \right)^{ - 1} \hfill \\ \sigma_{y} \left( x \right) = - P_{0} \left\{ {\left( {1 + \mu } \right)\left[ {1 - \frac{{x_{1} }}{a}\tan^{ - 1} \left( {\frac{a}{{x_{1} }}} \right)} \right] + \frac{1}{2}\left( {1 + \frac{{x_{1}^{2} }}{ab}} \right)^{ - 1} } \right\} \hfill \\ \sigma_{z} \left( x \right) = - P_{0} \left\{ {\left( {1 + \mu } \right)\left[ {1 - \frac{{x_{1} }}{b}\tan^{ - 1} \left( {\frac{b}{{x_{1} }}} \right)} \right] + \frac{1}{2}\left( {1 + \frac{{x_{1}^{2} }}{ab}} \right)^{ - 1} } \right\} \hfill \\ \end{gathered} \right. $$

where *σ*_*x*_ (*x*) is the elastic stress along X-direction, *P*_0_ is the maximum pressure at the center of the contact surface, *x*_1_ is the extrusion depth of the rolling ball, *a* is the major axis in the ellipse of the contact surface,* b* is the minor axis in the ellipse, *σ*_*y*_ (*x*) is the elastic stress along Y-direction,* μ* is Poisson's ratio, *σ*_*z*_ (*x*) is the elastic stress along Z-direction.

According to the maximum principal shear stress theory^33^, the Von-Mises equivalent stress is shown in Eq. (29).

29 $$ \sigma = \sqrt {\frac{{\left( {\sigma_{x} - \sigma_{y} } \right)^{2} + \left( {\sigma_{y} - \sigma_{z} } \right)^{2} + \left( {\sigma_{z} - \sigma_{x} } \right)^{2} }}{2}} $$

where *σ* is the Von-Mises equivalent stress, *σ*_*x*_ (*x*) is the elastic stress along X-direction, *σ*_*y*_ (*x*) is the elastic stress along Y-direction, *σ*_*z*_ (*x*) is the elastic stress along Z-direction.

The elastic strain components in the three directions are shown in Eq. (30). The calculation of the equivalence is shown in Eq. (31)^34^.

30 $$ \left\{ \begin{gathered} \varepsilon_{x} \left( x \right) = \frac{{\left[ {\sigma_{x} \left( x \right) - \mu \left( {\sigma_{y} \left( x \right) + \sigma_{z} \left( x \right)} \right)} \right]}}{{E_{2} }} \hfill \\ \varepsilon_{y} \left( x \right) = \frac{{\left[ {\sigma_{y} \left( x \right) - \mu \left( {\sigma_{x} \left( x \right) + \sigma_{z} \left( x \right)} \right)} \right]}}{{E_{2} }} \hfill \\ \varepsilon_{z} \left( x \right) = \frac{{\left[ {\sigma_{z} \left( x \right) - \mu \left( {\sigma_{x} \left( x \right) + \sigma_{y} \left( x \right)} \right)} \right]}}{{E_{2} }} \hfill \\ \end{gathered} \right. $$

31 $$ \varepsilon = \frac{\sigma }{{E_{2} }} $$

where *ε*_*x*_ (*x*) is the elastic strain along X-direction, *σ*_*x*_ (*x*) is the elastic stress along X-direction,* μ* is Poisson's ratio, *σ*_*y*_ (*x*) is the elastic stress along Y-direction, *σ*_*z*_ (*x*) is the elastic stress along Z-direction, *σ* is the Von-Mises equivalent stress, *E*_2_ is the elastic modulus of 42CrMo steel.

Except for elastic strain, the workpiece is plastically deformed with the effect of USRP. In general, the plastic deformation produced by the workpiece can be represented by the constitutive equation of the material or the work hardening model. At the same time, Neuber’s theory can also express the relationship between plastic stress and strain, where Neuber’s theorem states that plastic strain energy is equal to pseudo-strain energy, where Neuber’s theory^35^ is shown in Eq. (32).

32 $$ \sigma_{p} \varepsilon_{p} = \sigma_{e} \varepsilon_{e} $$

where *σ*_*p*_ is the plastic stress, *ε*_*p*_ is the plastic strain, *σ*_*e*_ is the elastic stress, and *ε*_*e*_ is the elastic strain.

According to the principle of USRP work hardening, the plastic strain rate of the material will be greatly affected by the ultrasonic high-frequency vibration frequency, the calculation of the plastic strain rate is shown in Eq. (33).

33 $$ \dot{\varepsilon } = \frac{d\varepsilon }{{dt}} = \frac{{dl_{m} }}{{l_{m} dt}} = \frac{{v_{m} }}{{l_{m} }} = \frac{{2\pi f_{r} A}}{A} = 2\pi f_{r} $$

where $\dot{\varepsilon }$ is the plastic strain rate, d*ε* / d*t* is the partial derivative of the time, d*l*_*m*_ / d*t* is the partial derivative of the time, *v*_*m*_ is the maximum vibration velocity, *l*_*m*_ is the maximum indentation of the rolling ball, *f*_*r*_ is the vibration frequency, *A* is the vibration amplitude.

During ultrasonic surface rolling process, the rolling ball applies a certain static pressure and vibrates at a specific frequency on the surface of 42CrMo steel, resulting in elastic–plastic deformation of the surface of material. In the elastic phase, the stress–strain relationship of the material follows the generalized Hooke’s law. When it exceeds the yield limit of material, the relationship between stress and strain is shown in Eq. (34)^36^.

34 $$ \sigma = A_{s} + B_{s} \varepsilon^{n} $$

where *σ* is the equivalent stress, *ε* is the equivalent plastic strain, *A*_*s*_ is the yield stress, *B*_*s*_ is the strain hardening coefficient, and *n* is the strain hardening exponent. When the yield stress of 42CrMo steel is known at high strain rates, the stress values corresponding to the strain of material can be determined. The magnitude of the static pressure determines the strength and stiffness of the workpiece. When the static pressure applied by USRP tool head reaches the plastic deformation stage of the material, although the overall shape may change, the volume remains unchanged. The surface of the workpiece undergoes changes in its crystallographic structure and grain composition when subjected to external forces. Grain slip occurs, and the distribution of plastic deformation within the metal body is non-uniform. After the external force is removed, different parts of the material exhibit varying degrees of elastic recovery. This results in the generation of internal stresses that balance each other between different parts of the material. This leads to a significant increase in residual stresses and hardness in the wind turbine bearing ring. Therefore, the proper selection of the static pressure can enhance the performance of the precision bearing ring after strengthening, such as load-bearing capacity, wear resistance, and fatigue strength.

### USRP strengthens the micromachining mechanism

**Figure 3:**
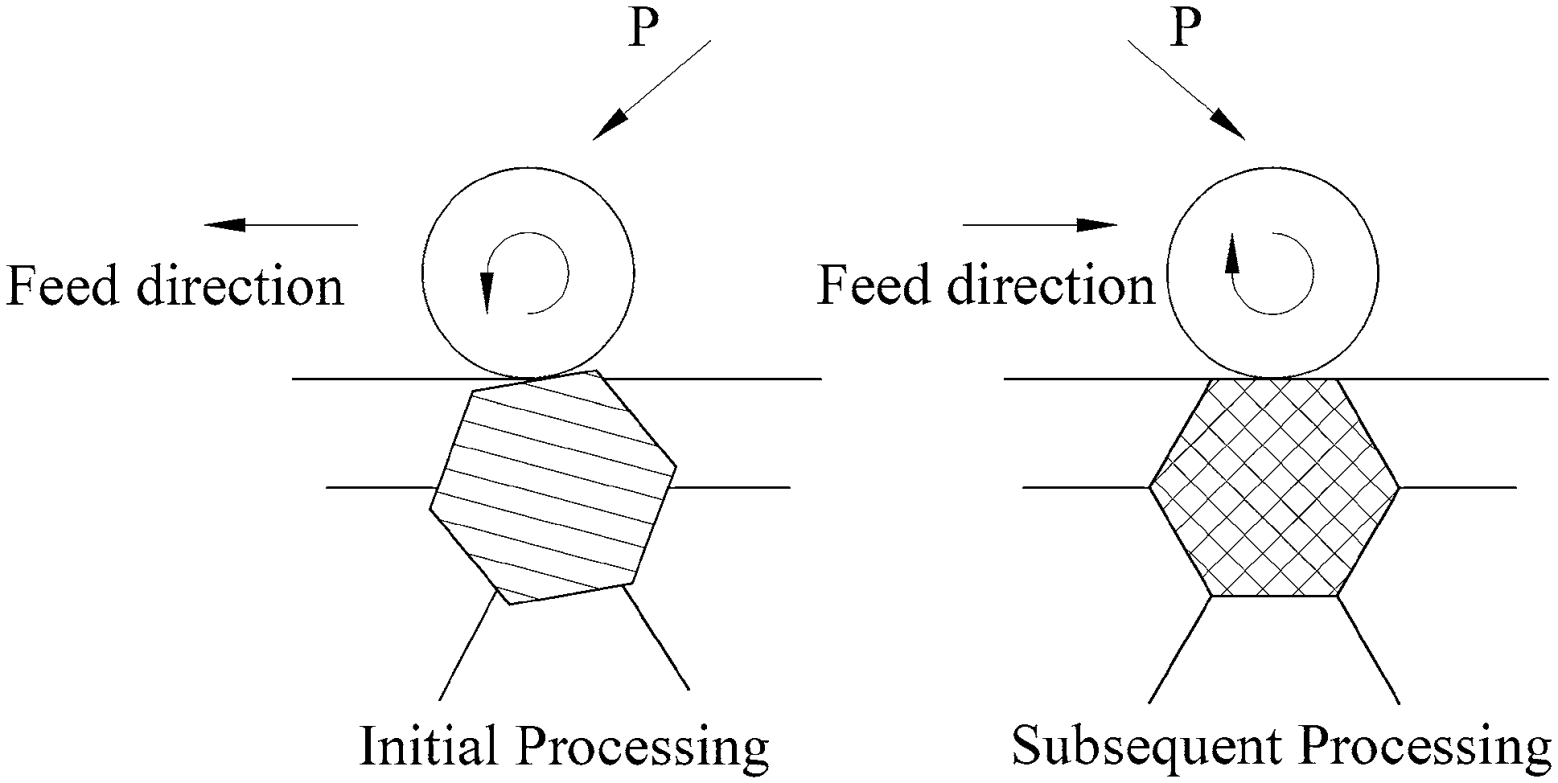
Plastic deformation of microstructural units.

During USRP strengthening, the rolling ball applies cyclic external loads repetitively to the surface of material. This repeated loading near the surface of the workpiece generates a stress field with a certain depth of influence. Within this stress field, small structural units experience plastic deformation, as shown in Fig. 3.

The rolling ball continuously impacts the workpiece, applying a sustained external load. Due to the workpiece's rotation and the feeding motion of the rolling ball, subsequent external loads act on different locations of the surface of workpiece. Each impact generates a new stress field, leading to changes in the direction of plastic deformation within the microstructural units in this new field. The phenomenon of grain refinement during the strengthening process results from the cumulative effect of plastic deformations. The extent of plastic strain increases as the strengthening process continues, expanding from the surface of the workpiece towards deeper layers. Moreover, the degree of plastic deformation intensifies. Microscopic plastic deformation comprises dislocation and twinning forms. During the strengthening process, the workpiece's surface experiences high strain and strain rates, along with a high dislocation density. These factors contribute to an increased degree of plastic deformation, thereby leading to the formation of a refined grain structure on the surface of workpiece, ultimately enhancing the performance of the workpiece.

During the ultrasonic rotary swaging process, the workpiece is simultaneously subjected to ultrasonic vibration stress and mechanical extrusion stress. The ultrasonic vibration stress generated inside the workpiece is described by the longitudinal wave propagation equation:

35 $$ u(x,t) = A(x)\sin (\varpi t) = A\cos \frac{{2\pi f_{r} }}{{c_{v} }}x\sin 2\pi f_{r} t $$

The internal stress due to workpiece vibration can be expressed as:

36 $$ \sigma = \varepsilon E = u(x,t)E = AE\frac{{2\pi f_{r} }}{{c_{v} }}\sin \frac{{2\pi f_{r} }}{{c_{v} }}x\sin 2\pi f_{r} t $$

where *c*_*v*_ represents the speed of sound wave propagation in the medium, *ρ* is the density of the workpiece material, and *E* stands for the elastic modulus of the workpiece. Therefore, the maximum internal stress due to vibration is calculated as follows:

37 $$ \sigma_{\max } = AE\frac{{2\pi f_{r} }}{{c_{v} }} = 2\pi f_{r} A\sqrt {E\rho } $$

The vibrational stress generated by ultrasonic frequency oscillations counteracts the pre-existing internal stresses within the workpiece. The hardness retained by the workpiece after vibration loading serves as the equivalent hardness of the workpiece material. However, due to the influence of vibrational internal stress, the equivalent hardness of the workpiece material is significantly reduced, resulting in a phenomenon known as ultrasonic softening. As indicated by the above equation, the magnitude of vibrational internal stress is directly proportional to the amplitude and frequency of the vibration. In the process of ultrasonic vibration transmission through the workpiece material, the frequency remains constant, while the amplitude gradually decreases from the workpiece surface towards its interior. Consequently, the degree of ultrasonic softening in the workpiece material diminishes progressively from the surface to the interior. During the ultrasonic strengthening process, this is reflected in a gradual reduction in the extent of grain refinement in the workpiece material, ultimately reaching a stable state.

**Figure 4:**
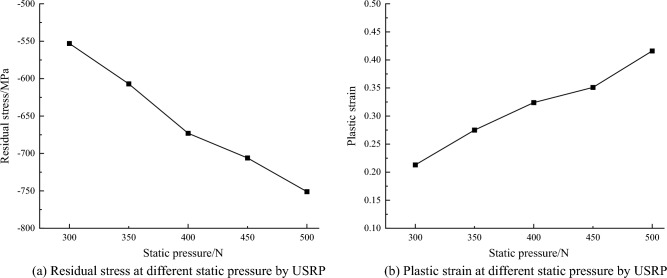
Theoretical calculation at different static pressure by USRP.

When the elastic modulus of 42CrMo steel is 200 GPa, the material density is 7850 kg/m^3^, the static pressures are 300N, 350N, 400N, 450N, and 500N respectively, the amplitude is 5 μm and the ultrasonic vibration frequency is 20 kHz, the material property and process parameters are substituted into the above equations to obtain theoretical values of residual stress and plastic strain under different static pressures. As seen from Fig. 4a, with the increasing static pressure, the residual stresses gradually increase. When the static pressure is 500N, the maximum residual stress is reached, which amounts to − 751 MPa. This is because as the static pressure increases, the material's plastic deformation intensifies. Under the impact of ultrasonic waves and high-frequency vibration, the crystal grains on the surface of the part noticeably refine, leading to an increase in residual stresses. As shown in Fig. 4b, while keeping other process parameters constant, the plastic strain of USRP under static pressures ranging from 300 to 500N is depicted. The changing trend of plastic strain aligns with that of residual stress. With an increase in static pressure, the plastic strain also increases. When the static pressure reaches 500N, the plastic strain reaches its maximum, measuring 0.416. As static pressure increases, the contact stress applied to the part's surface rises, leading to an increase in plastic strain.

## USRP experiment

### Materials

The experimental workpiece used unquenched 42CrMo steel bar stock, which the diameter is 30 mm, the length is 300 mm, the initial roughness of 1.11 μm and the initial Rockwell hardness is 24.3HRC. The chemical composition of 42CrMo steel is shown in Table 1.

**Table 1:** The chemical composition of 42CrMo steel.

Fe	C	Cr	Mo	Mn	Si	Ni
97.44%	0.37%	0.98%	0.21%	0.77%	0.15%	0.04%

The single-factor experimental method is used for 42CrMo steel by USRP, and the processing parameters are shown in the Table 2.

**Table 2:** The process parameter of USRP.

NO	Workpiece speed/r/min	Feed rate/mm/min	Static pressure/N	Amplitude/μm
1	350	15	300	5
2	350	15	350	5
3	350	15	400	5
4	350	15	450	5
5	350	15	500	5

### Methods

**Figure 5:**
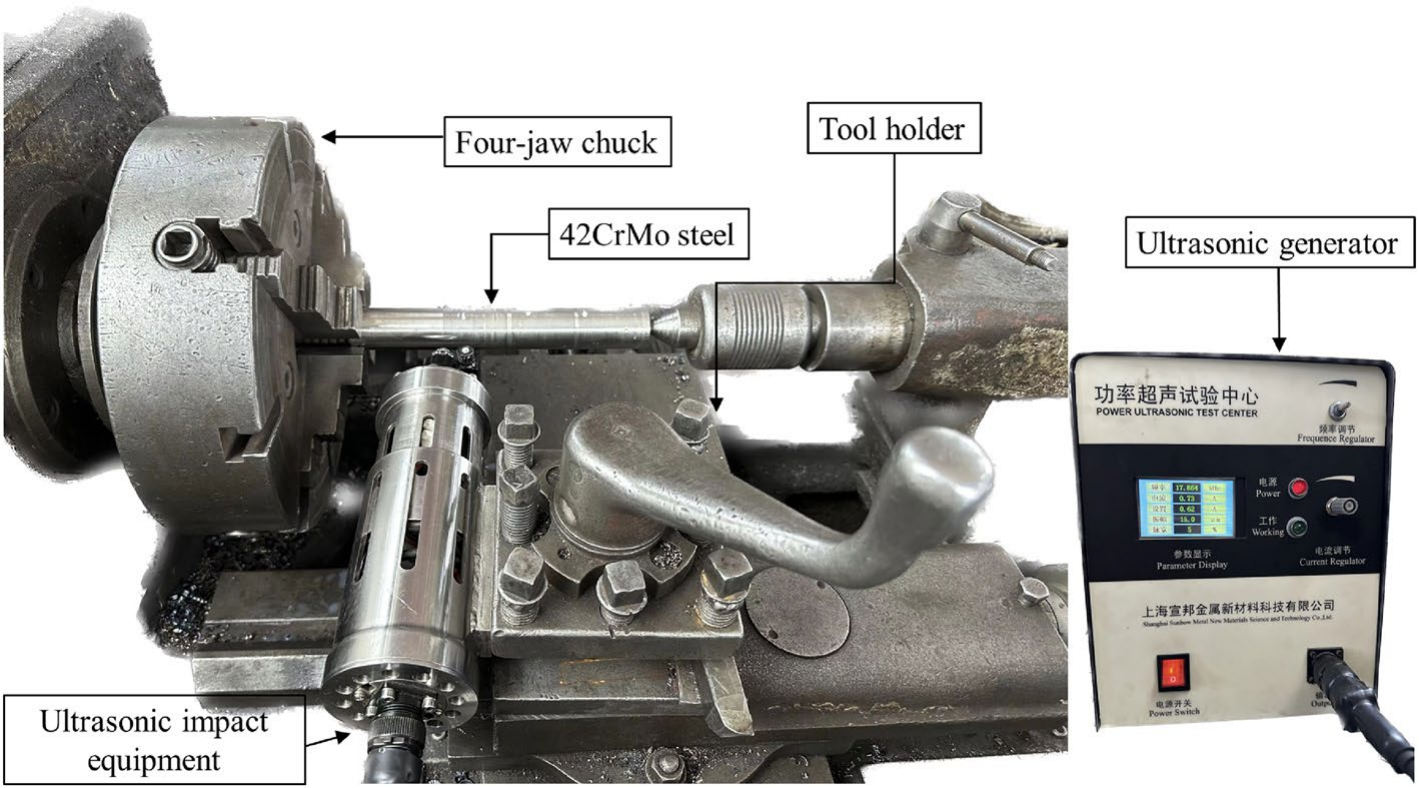
USRP test processing diagram.

As shown in Fig. 5, the USRP experimental diagram for 42CrMo steel is displayed. 42CrMo steel bar was processed by USRP method, which the test was carried out on CD6140A lathe produced by Dalian Machine Tool Group. Each set of processing parameters was tested using a single bar in the USRP experiments. The length of the processing area for each test group was 50 mm. Simultaneously, the first experimental region in the group and the second experimental region were spaced 5 mm apart along the axial direction of the bar, and so on, with a 5 mm gap between the fourth and fifth experimental regions. To reduce measurement errors, hardness and surface roughness were measured at five different positions within each segment of the processing area, and the averages were calculated to minimize measurement discrepancies. After all the parts are processed, the wire EDM machine was used to cut and sample each section of the area, and 5 workpieces are obtained. In order to be able to observe the metallographic structure of the material, 500 mesh, 800 mesh, 1000 mesh, 1500 mesh, 2000 mesh water sandpaper are used to grind the section of the part on the metallographic pre-grinding machine, and then polished in the metallographic polishing machine, and W4.0 diamond polishing paste is evenly applied on the polishing cloth in order to achieve the mirror effect. At the same time, the surface of the specimen is washed with an alcohol solution to prevent the parts from rusting, and the surface is dried with the hair dryer. Finally, diluted 4% nitric acid alcohol solution was used to corrode the surface of the part, and when the section of the part appears foggy, the corrosion is stopped, and the corrosion time can be 8–10 s. The SF-150A Rockwell hardness tester produced by Changzhou Sanfeng Company is used to measure the surface hardness of parts. Because the sample tested was 42CrMo steel cylinder bar, whose outer contour was not flat and its surface roughness could not be measured on 3D topography instrument, so the surface roughness was measured by MarSurf VD280 surface profiler produced by Marl. The LEXT OLS5100 3D laser surface topography instrument produced by OLYMPUS was used to measure its surface micromorphology. At the same time, the VEGA3 tungsten filament scanning electron microscope produced by TESCAN was used to photograph the microstructure of the corroded parts.

### Results

**Figure 6:**
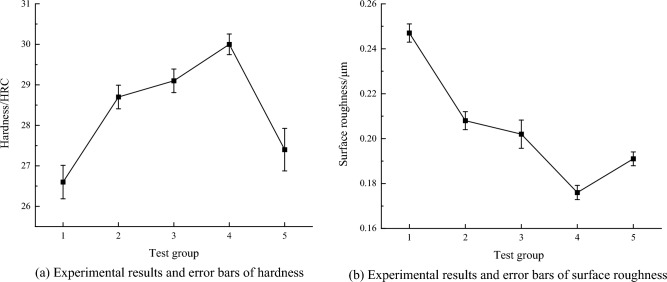
The results of the surface performance by USRP.

The results of the five sets of single-factor USRP tests performed are shown in Fig. 6, from which it can be obtained that when the static pressure increases from 300 to 500N, the Rockwell hardness increases from 20 to 30HRC, and when the static pressure is located at 450N, the hardness reaches to the maximum. With the increase of static pressure, the degree of plastic deformation intensifies, the degree of work hardening increases significantly, in the view of increase of surface hardness, it can clearly show that the phenomenon of the work hardening occurs, grains are significantly refined, dislocation slip movement mode intensifies so that the surface hardness of 42CrMo steel significantly increases, at the same time, it can be seen that the hardness does not increase with the increase of static pressure, when the static pressure increases to 500N, The hardness value decreased from 30HRC to 27.4HRC, which indicates that the degree of plastic deformation has reached the limit, and the grain boundaries, subgrain boundaries and refined grains formed inside 42CrMo steel under the action of USRP can prevent further deformation of the material, while the increased static pressure will destroy the newly formed subgrain boundaries, so that the grain boundaries are destroyed, thereby reducing the surface hardness of its parts. It can be seen that choosing the right static pressure is very important. Meanwhile, it can be seen that with the increase of static pressure, the surface roughness decreases from 0.247 μm to 0.176 μm, and when the static pressure is 450N, the surface roughness reaches the minimum. The static pressure is too low to flatten the knife marks in that the last process of 42CrMo steel bar is the fine turning. As long as the impact of the rolling ball on the part is large enough to flatten the uneven knife marks, the surface roughness can be reduced. When the static pressure increases to 500N, the surface roughness increases to 0.191 μm. When the static pressure is too large, the vibration effect of the ultrasonic rolling gun intensifies, which increases the disturbance of the entire ultrasonic device, but weakens the stability of the machine tool, so when the rolling ball is rolled axially along the part, the rolling ball jumps up and down to cause the surface roughness to increase.

**Figure 7:**
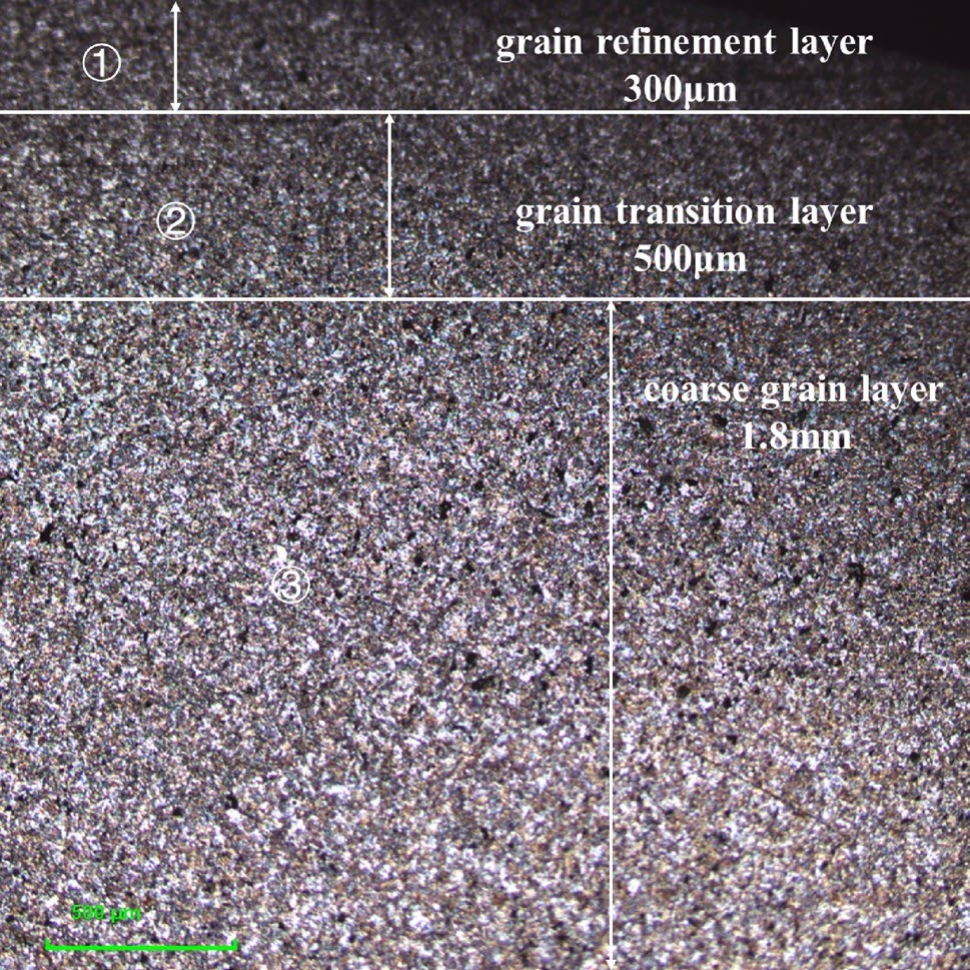
Microstructure of the surface layer of 42CrMo steel treated by USRP.

As shown in Fig. 7, the microstructure diagram of the surface section of the processed 42CrMo steel is displayed. It can be seen that the microstructure of 42CrMo steel rod without quenching is a mixture of degenerated pearlite and ferrite. Among them, the pearlite type is flaky pearlite. After USRP treatment, the microstructure of 42CrMo steel along the rolling direction is elongated, and the depth of the grain refinement layer is about 300 μm. A situation that is significantly different from the original texture. The metallographic structure near the surface of the material is finer, elongated in the rolling direction, and the grain size is small. While the grain inside the material far from the surface of the material is coarse, the grain size is large, and the parts before USRP treatment have no obvious change. It can be seen from Fig. 7 that 42CrMo steel section treated by USRP can be divided into three parts, the first part is the surface: grain refinement layer, the thickness of the refinement layer is about 300 μm; The second part is the grain transition layer; The third part is the coarse grain layer. Since the parts are not quenched, the structure present in the parts is a mixture of pearlite and ferrite, and no structures such as martensite appear.

This is due to the dislocation phenomenon inside the part after USRP treatment, under the combined action of static pressure and high-frequency vibration, two dislocation forms of motion occur on the surface of the material: slippage and climbing movement. Among them, slip refers to the phenomenon of slip under the action of shear stress, while climbing refers to movement in the direction perpendicular to the slip surface. The surface of USRP reinforced part is Frank Read dislocation mechanism. It refers to the phenomenon of dislocation entanglement between grains and grains under these two forms of motion, and new dislocation walls are constantly generated and formed, hindering the relative work between grains. At the same time, new dislocation walls are formed between the grains, and the grains split into smaller grains, forming grain boundaries at smaller angles. Under the hindrance of the small angle grain boundary, it will make it more difficult for the part to undergo plastic deformation, so that the large grain is transformed into a small grain, and the surface performance of the part is improved. In order to strengthen the surface of the part, the part is prevented from peeling or even breaking by hindering the dislocation movement. USRP enhanced part surface mechanism is to prefabricate more dislocations inside the part, and hinder the occurrence of dislocation motion by adding initial dislocations. Simultaneously, more grain boundaries are split to produce small angle grain boundaries, thereby refining the grain and enhancing the surface performance of the part.

**Figure 8:**
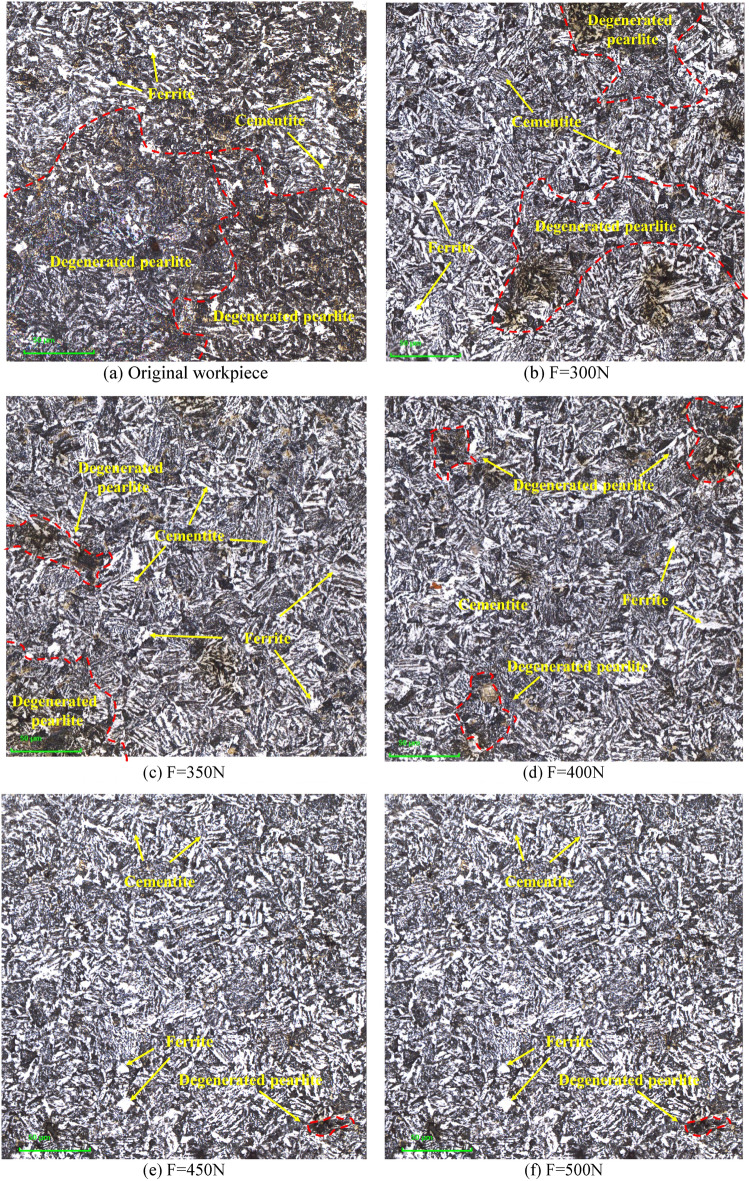
Original and USRP-Treated microstructure at 1000 × magnification in CLSM.

Figure 8 shows the microstructure of untreated and 5 groups of specimens measured with 1000 × magnification at a depth of 300 μm from the surface layer under the LEXT OLS5100 Confocal Laser Scanning Microscopy (CLSM). Because cementite is strongly resistant to corrosion by nitric acid alcohol solution, so it is displayed as white microstructure under the three-dimensional topography instrument. Figure 8a shows the microstructure of 42CrMo steel without USRP test, it can be seen that the internal structure of the material without ultrasonic vibration treatment is scattered in the basal corpuscle, and its metallographic structure is completely consistent with the state of the material when it leaves the factory, whose distribution is scattered, and there is no regular fixed shape. Figure 8b–f show the microstructure inside 42CrMo steel matrix when the static pressure is 300N, 350N, 400N, 450N and 500N respectively, feed rate is 15 mm/min, workpiece speed is 300 r/min and amplitude is 5 μm. It can be seen that cementite is distributed more in 42CrMo steel, and it is scattered in the material matrix in a "needle-like" manner. Among them, the black structure is pearlite, which is composed of ferrite and cementite. Because the ferrite in pearlite is mainly laminated, cementite is also arranged in layers, and cementite and ferrite overlap each other, because the junction is most susceptible to corrosion under the corrosion of nitric acid alcohol, and the metallographic color changes after corrosion, so the cementite in pearlite shows black. The ferrite is distributed between cementite in the shape of a "short rod", and the amount of cementite in pearlite is significantly less than that of ferrite, so the ferrite of the sheet structure is much thicker than that of the thin layer pearlite. It can be seen that the metallographic size of the workpiece treated by USRP is significantly smaller than the microstructure size of the specimen without USRP. This shows that under the action of USRP, the grain size of the surface layer of 42CrMo steel is refined, and the cementite size is reduced. Ferrite in pearlite is crushed under the action of high-frequency vibration shock and static pressure, and the volume is reduced. At the same time, it can be clearly seen that the microstructure of 42CrMo steel is elongated along the rolling direction. However, the metallographic structure inside the matrix is weakly affected by USRP, and the ultrasonic energy along the depth of the layer cannot reach the inside of the matrix, so the metallographic size is large. However, USRP mainly improves the surface performance of the part, and its internal metallographic structure and grain changes do not affect the service performance of the part.

**Figure 9:**
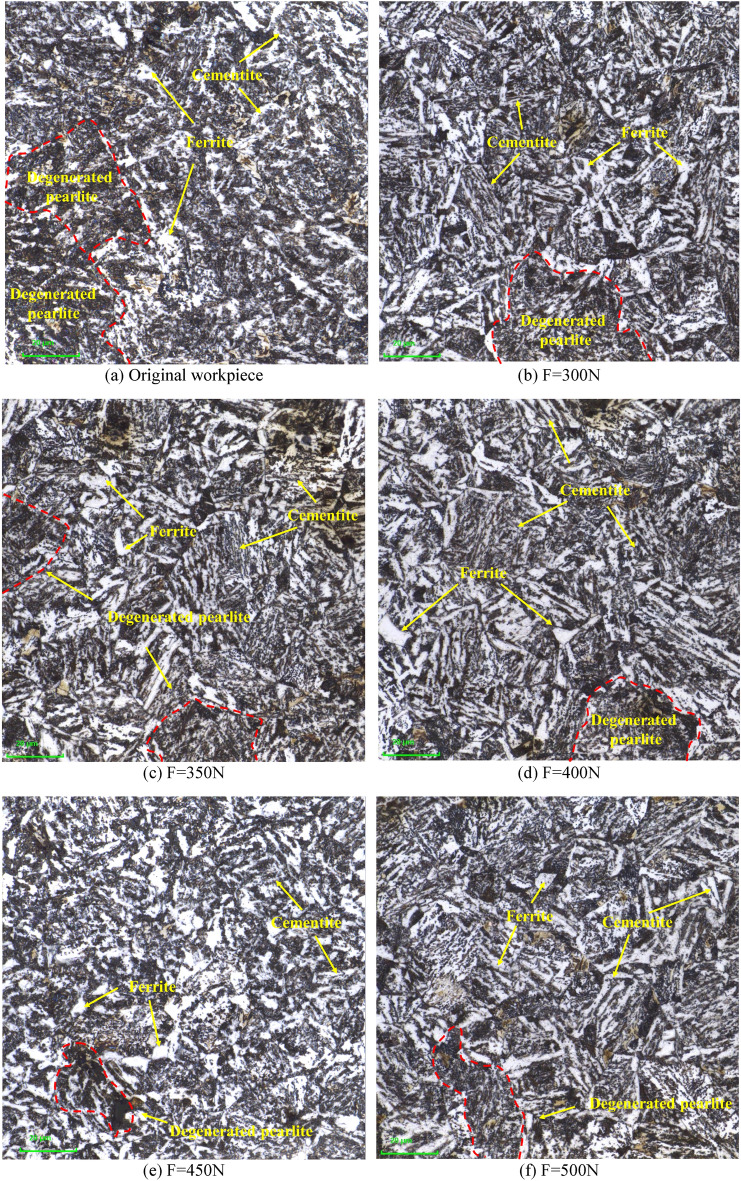
Original and USRP-Treated microstructure at 2000 × magnification in CLSM.

Figure 9 shows the microstructure diagram of non-USRP and USRP at a depth of 300 μm from the surface layer with 2000 × magnification in CLSM. Figure 9a shows the microstructure of original 42CrMo steel without USRP test. At the same time, it can be seen that the microstructure of Fig. 9a is significantly different from that of Fig. 9b–f after USRP treatment, and the content of the ferrite in black pearlite is obviously more than that of the internal structure of the USRP-treated material. Moreover, the black lumpy ferrite is glued together. Figure 9b–e shows the metallographic structure at 2000X magnification of in the LEXT OLS5100 3D laser surface topography instrument under five sets of parameters, because the imaging principle of the optical microscope is that imaging is performed by the reflection of light. Therefore, when the material is corroded, the flatter area will be brighter under the optical microscope.

Compared to Fig. 8, it is more obvious that the white cementite is arranged parallel between the cementite. At the same time, ferrite and long strips of cementite together constitute degenerated pearlite. The long strip cementite contains more inside 42CrMo steel. The black pearlite undergoes fragmentation under the action of USRP, and the volume in the pearlite tissue decreases. Inside the parts treated by USRP, regular degenerated pearlite is produced, while the grain size is significantly reduced, the layered spacing of the lamellar pearlite is significantly reduced, its ability to resist plastic deformation is enhanced, and the hindrance to dislocation movement is increased, thereby significantly improving the strength and hardness of the part. Because degenerated pearlite is made up of ferrite and cementite. In other words, after USRP treatment, ultrasonic impact will crush the ferrite, so that cracks and gaps will occur inside the ferrite, and the large ferrite will be split into small ferrite, and the cracks will develop along the direction of cementite, so that the contact area of the ferrite increases, which increases the strength and toughness of the material, thereby enhancing the wear resistance of the part, which is also the reason why USRP enhances the surface performance of the part.

**Figure 10:**
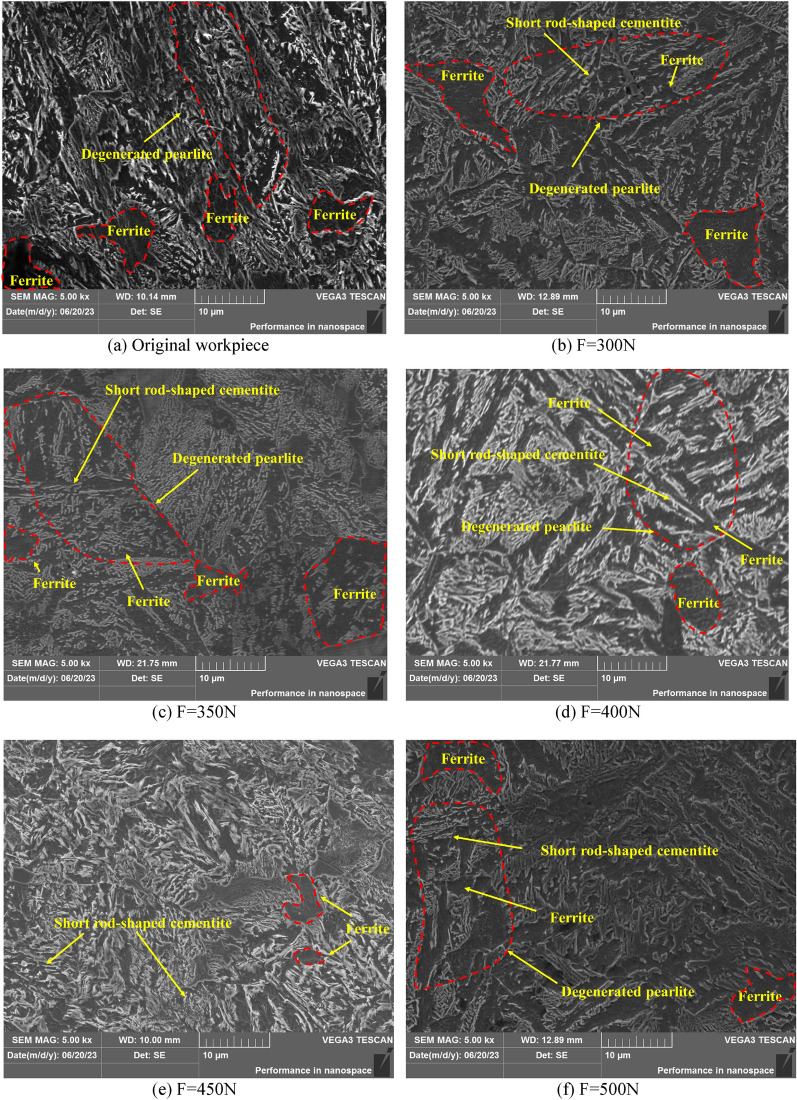
Original and USRP-Treated microstructure at 5000 × magnification in SEM.

In order to further observe the microstructure of 42CrMo steel under the action of USRP, the VEGA3 tungsten filament scanning electron microscope was used to measure the sample. Figure 10 shows the microstructure diagram of 42CrMo steel after USRP treatment and untreated USRP. It can be seen that the microstructure at a depth of 300 μm from the surface layer with 5000 × magnification is mainly pearlite, which is consist of the ferrite and the cementite. Figure 10a shows the 42CrMo steel matrix without USRP treatment, the original microstructure has a large number of ferrites, which is arranged in parallel, this large block of ferrite will lead to better plasticity and toughness of 42CrMo steel, but lower strength and hardness. The mechanism of action of USRP reinforced 42CrMo steel is to crush the bulk ferrite under the high-frequency oscillation impact, and distribute it in the matrix after separating and dispersing, thereby strengthening the part itself. Figure 10b shows the microstructure when the amplitude is 5 μm, the static pressure is 300N, the workpiece speed is 350r/min, and the feed speed is 15 mm/min, it can be seen that the number of ferrites has decreased, and the volume has become smaller, and the ferrite is squeezed around the cementite under the action of USRP and arranged in gaps. However, at the same time, it can also be seen that there are two large ferrites in the upper left and lower right corners of the figure, indicating that the crushing effect of USRP on ferrite is not sufficient. The circled red dotted line is degenerated pearlite, of which the black block is ferrite, and the short white bar is cementite. Degenerated pearlite is expressed as a mixture of short rod-shaped cementite and ferrite obtained by incorporating carbon into γ-Fe and undergoing simultaneous co-analysis transformation. The amount of pearlite in 42CrMo steel is not less. Figure 10c–f show the metallographic structure diagram when the static pressure increases from 350 to 500N with the amplitude, workpiece speed, and feed rate remain unchanged. The cementite in Fig. 10c is clustered together under USRP, while the ferrite is squeezed flat around the cementite under USRP. The microstructure shown in Fig. 10d is more visible than the microstructure captured by the surface topography instrument, the black ferrite is broken under the action of USRP, and like the cementite, it is distributed in a strip shape in the matrix, As shown in Fig. 10e,f, the ferrite in the microstructure is further fragmented under the action of USRP, and the microstructure morphology is significantly reduced. The white cementite is scattered in the matrix, and the cementite and ferrite are alternately arranged in the structure. Compared with Fig. 10e, when the static pressure is 500N and other process parameters stay the same, Fig. 10f shows that the extent of the improvement of microstructure refinement is not obvious in that under the action of USRP the material over-extrusion phenomenon occurs, destroying the newly formed subgrain boundary, resulting in 42CrMo steel strength and hardness decrease, which is consistent with the hardness value measured by the Rockwell apparatus, which the hardness first increases and then decreases.

**Figure 11:**
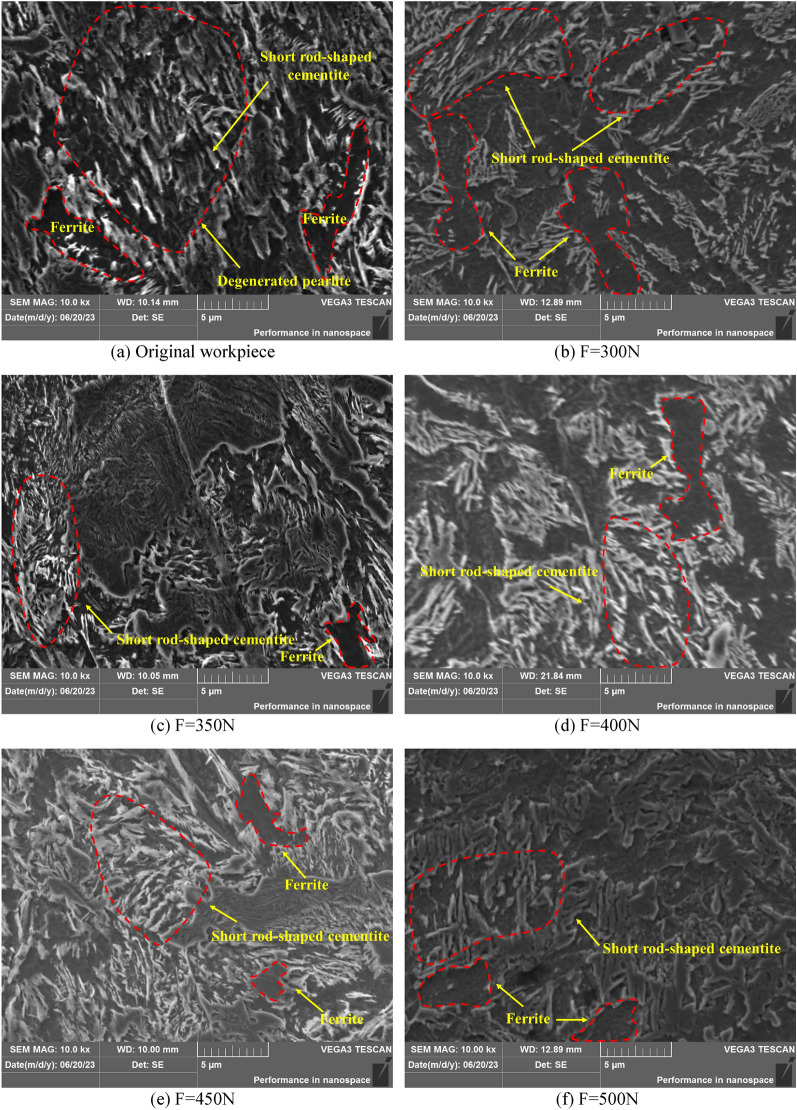
Original and USRP-Treated microstructure at 10,000 × magnification in SEM.

Figure 11 shows the microstructure of 42CrMo steel that has not been USRP and tested by USRP under a tungsten filament SEM at a depth of 300 μm from the surface layer with 10,000 × magnification. Since the imaging principle of SEM is different from OM, its detection principle is to generate electronic images on the computer by emitting an electronic signal to the surface of the material and receiving the physical signal through the receiver. Therefore, the peaks of the bulge on the surface of the material appear bright. Therefore, the white bright stripes in the figure are cementite. When it is at 10,000 × magnification, the microstructure is displayed more clearly. the metallographic structure of 42CrMo steel without USRP treatment is shown in Fig. 11a, with ferrite distributed in the center of the matrix. Compared with Fig. 11b–f, the white cementite is densely distributed, the ferrite is distributed in the matrix in a “cashew type”, the spacing between ferrite and cementite is small in 42CrMo steel without USRP, the volume of ferrite is larger, and the initial mechanical properties of 42CrMo steel are weaker than the specimens treated by USRP. As shown in Fig. 11b,c, when the amplitude, workpiece speed, and feed rate is unchanged, and the static pressure is located at 300N and 350N, respectively, the microstructure diagram is displayed. USRP causes the ferrite in pearlite to break up, splitting the large block of ferrite into narrow and long ferrite, so that it is cross-arranged and distributed in 42CrMo steel. As shown in Fig. 11d, it is the microstructure diagram when the workpiece speed is 350r/min, the feed rate is 15 mm/min, the amplitude is 5 μm, and the static pressure is 400N. Compared with Fig. 11a,b, the morphology of the metallographic structure gradually decreases, and the ferrite is separated by cementite one by one, showing a feathery distribution in the matrix, while the ferrite and cementite are alternately arranged to form a degenerated pearlite. As shown in Fig. 11e,f, the amplitude, workpiece speed and feed rate remain unchanged, and the static pressure is 450N and 500N respectively. It can be clearly seen that with the increase of static pressure, whose microstructure morphology is further divided, compared with Fig. 11a–d, the amount of cementite in 42CrMo steel microstructure increases, while the volume of ferrite decreases, the gap is arranged around the cementite, this alternating combination form of large ambassador parts strength, hardness and even toughness as much as possible, USRP has a positive effect on improving the microstructure of parts. However, As shown in Fig. 11f, the improvement of the microstructure refinement in 42CrMo steel under the action of USRP is not obvious. When the static pressure is 450N. and the ferrite splits from the large block shape to small shapes, and the interval is arranged around the ferrite, the metallographic structure is evenly distributed at this time, thereby significantly improving the surface performance of 42CrMo steel.

## Discussion

From the above analysis, it can be seen that the USRP strengthening part mechanism is mainly through high-frequency vibration and static pressure to shape and deform the internal parts and form dislocation entanglements. As the degree of deformation increases, dislocation multiplication occurs. Position changes between different grains produce new cell-like tissues by dislocation proliferation. Under the action of high-frequency vibration of the rolling ball, the deformation degree is further increased, and the formed cell-like tissue is split into subcrystalline grains, and under the action of USRP, the subcrystalline grains are further transformed into small-angle grain boundaries, so that the dislocations per unit area and even grain boundaries become more, resulting in more difficult deformation between grains and grains, thereby improving the strength, hardness and wear resistance of parts. Through the analysis provided above, it is evident that there exists a high level of consistency between the experimental outcomes of USRP and the theoretical predictions.

Because the atomic ordering of common polycrystalline materials is not regularly arranged, there are often two forms: spiral dislocation and blade dislocation. The movement direction of the blade dislocation is parallel to the crystal slip direction, while the crystal slip direction of the blade dislocation is 90 degrees to the direction of motion. The material continues to produce dislocation and even dislocation entanglement in the existing crystal defects, which further increases the degree of difficult deformation of grain boundaries. Plastic deformation is mainly carried out in two forms: twin and dislocation slip. Among them, slip refers to the slow movement of dislocation lines on the slip surface containing the Bergs vector under the action of shear stress, similar to earthworm crawling, resulting in permanent deformation of grains on the surface of 42CrMo steel. However, twin deformation refers to the process by which a specific crystal plane atom of a polycrystalline material undergoes shear strain in a certain direction. At the same time, the twin atoms and the non-occurring atoms form mirror symmetry through the twin face, which is another important shaping and deformation mechanism in addition to the phenomenon of dislocation-slip. It is precisely the plastic deformation that occurs in USRP under these two deformation mechanisms. 42CrMo steel, as a body-centered cubic structure (BBC) metal, has 48 slip systems. Under the action of shear stress, the phenomenon of dislocation slip occurs. In general, the lower the layer fault, the more likely twins are to occur. When the delamination energy increases, the plastic deformation is carried out in the form of dislocation. Then for 42CrMo steel, its layer fault energy is very high, which shows that its plastic deformation method is mainly dislocation. USRP reinforced 42CrMo steel obtains a refined grain layer by producing severe shaping deformation on the surface of the material. It is under the combined action of high-frequency vibration and static pressure that the intermolecular force increases, resulting in dislocation slip inside the material. For 42CrMo steel with high dislocation energy, its grain refinement process is developed into four steps: (1) dislocation value-added; (2) Dislocation lines and dislocation entanglements; (3) The matrix grain is refined into subcrystalline grain; (4) New grains are formed under dynamic recrystallization.

Because the critical shear stress that needs to be overcome when the metal dislocation slips of the body-centered cubic structure is not large, the slip surface and slip direction are prone to movement. 42CrMo steel crystals often have pores, impurities and other defects inside. Under the superposition of high-frequency vibration and static pressure, the defect form and atomic arrangement inside the metal change, and under the intensification of the strain of the material, the atomic arrangement and defect form change greater, so that the dislocation proliferates the whole material face, at the same time, the original coarse grain in 42CrMo steel produces dislocation lines. The atomic arrangement structure and surface arrangement inside the metal are different, the surface is used as the end surface of the atomic arrangement, and there is no atomic bond connected to it outside the surface, so the coordination number of the metal surface is smaller than the coordination number inside the metal, so that the atomic energy of the metal surface is greater than the metal interior. Therefore, the physical properties and chemical properties of the 42CrMo steel surface are very active, and plastic deformation is more likely to occur under the action of USRP.

At the same time, the initial grain of 42CrMo steel continues to slip and proliferate increasingly, and the motion between the grain and the grain causes the dislocation line to be continuously concentrated, resulting in the phenomenon of dislocation wall and dislocation entanglement, and the initial grain is separated by the dislocation wall and dislocation entanglement to form dislocation cells with small morphological size. Under the continuous action of increasing the degree of strain, the dislocation density increases. In order to achieve equilibrium of the energy inside the metal, dislocation walls and dislocation entanglements with high dislocation density disappear and rearrange, resulting in subgrain boundaries at low angles. The dislocation density decreases under the action of subgrain boundaries, thus bringing the microscopic strain to equilibrium. USRP applied to the surface of 42CrMo steel continuously increases the degree of strain, and the subgrain boundary dislocation under the low angle grain boundary is continuously updated and replaced, so that the orientation difference near the subgrain boundary continues to increase, and the arrangement of grains changes to random distribution. Once the rate at which the dislocation occurs and the rate at which it disappears are equal, the degree of change in strain no longer has an effect on the grain size, that is, the increase in strain no longer leads to a smaller grain size. At this point, the metal internally reaches equilibrium and the grain size no longer changes.

The mechanism of grain refinement caused by USRP strengthening of the surface of 42CrMo steel is as follows: 42CrMo steel produces severe plastic deformation under the action of high-frequency vibration and static pressure of USRP. Dislocations inside the material produce climbing and slipping movements, and when the degree of dislocation reaches saturation, the original coarse grains are divided to form a large number of cell-like structures. In places where the dislocation density of dislocation walls and dislocation entanglements is large, dislocations continue to multiply, disappear and rearrange, so that the orientation difference between cell-like structures becomes larger, so that the original coarse grains are split into subcrystalline grains, and at the same time the dislocation walls and dislocations entanglement are transformed into low-angle grain boundaries. The large strain and large strain rate generated by plastic deformation, the coarse grain splits into sub-grains, and the process of dislocation entanglement into low-angle grain boundaries constantly occurs and updates, so that the original grain of 42CrMo steel is refined.

## Conclusions

This paper employed CLSM and SEM characterization methods, in conjunction with the principles of contact mechanics and Hertzian contact theory, to conduct an in-depth investigation into the mechanisms of strengthening 42CrMo steel using USRP and the patterns of microstructural changes. The following conclusions were drawn:

1. In USRP strengthening processing, the rolling ball can freely roll, significantly reducing the friction coefficient on the contact surface compared to extrusion processes. This reduction in lateral stress in the contact zone considerably lowers the generation of frictional heat, resulting in greater stability in surface quality.
2. The phenomenon of grain refinement during the strengthening process results from the cumulative effect of plastic deformations. The extent of plastic strain increases as the strengthening process continues, expanding from the surface of the workpiece towards deeper layers.
3. The distribution patterns of residual stress and plastic strain through theoretical analysis, which shows that with an increase in static pressure, residual stress and plastic strain gradually increase, the hardness firstly increases and then decreases, while surface roughness exhibits an initial decrease followed by an increase.
4. With the static pressure continues to increase, the number of ferrite phases inside 42CrMo steel after USRP treatment gradually decreases, and the size of carbide phases diminishes. The metallographic size of the workpiece treated by USRP is significantly smaller than the microstructure size of the specimen without USRP.

## Data Availability

The data that support the findings of this study are available on request from the corresponding author. The data are not publicly available due to privacy or ethical restrictions.
